# Accumulation of 3-Monochloro-Propanediol Esters in Kidney Tissues of Patients with Human Renal Cell Carcinoma

**DOI:** 10.3390/cancers16193313

**Published:** 2024-09-27

**Authors:** Che-Yuan Hu, Yu-An Wang, Kai-Wei Liao, Hung-Tsung Wu, Chien-Hui Ou, Choon Hui Tan, Wei-Ju Lee

**Affiliations:** 1 Department of Urology, National Cheng Kung University Hospital, College of Medicine, National Cheng Kung University, Tainan 701, Taiwan; 2 Master Program in Food Safety, College of Nutrition, Taipei Medical University, Taipei 110, Taiwan; 3 School of Food Safety, College of Nutrition, Taipei Medical University, Taipei 110, Taiwan; 4 Department of Internal Medicine, School of Medicine, College of Medicine, National Cheng Kung University, Tainan 701, Taiwan; 5 Department of Urology, Tainan Hospital, Ministry of Health and Welfare, Tainan 701, Taiwan; 6 Department of Food Science and Nutrition, Faculty of Applied Sciences, UCSI University, Kuala Lumpur 56000, Malaysia; tanch@ucsiuniversity.edu.my

**Keywords:** process contaminant, 3-MCPDEs, kidney, renal cell carcinoma, method validation

## Abstract

**Simple Summary:**

3-Monochloropropanediol esters (3-MCPDEs) are process contaminants generated during food processing. Although there is no clinical evidence directly linking them to adverse health effects, concerns have been raised regarding their potential to cause renal damage and cancer. To investigate this, we analyzed the concentrations of 3-MCPDEs in kidney tissues from 68 individuals, categorized into two groups: those with kidney cancer and those without. The levels of 3-MCPDEs were quantified using a validated analytical method, with the analyst blinded to the sample origins to ensure objectivity. The results indicated that 3-MCPDE concentrations were significantly higher in the kidneys of patients with kidney cancer compared to those without cancer. However, no significant correlation was found between 3-MCPDE levels and tumor size or stage. These findings suggest that 3-MCPDEs can accumulate in human kidneys and that their levels are elevated in cancerous tissues.

**Abstract:**

Background: 3-Monochloro-propanediol esters (3-MCPDEs), commonly found in refined edible oils and related products, have generated concerns due to their nephrotoxicity and carcinogenicity, yet clinical evidence remains limited. Objectives: In this study, we aimed to assess, for the first time, the accumulation of 3-MCPDEs in human kidney tissues, focusing on 68 participants, some with and others without renal cell carcinoma (RCC). Methods: An analytical method for 3-MCPDE determination in kidney tissues underwent partial validation to ensure its suitability for sample analysis. The analyst was blind to the sample groups. Results: Results revealed significantly higher 3-MCPDE levels in RCC patients compared to non-RCC counterparts (0.22 vs. 0.01 µg/g) (*p* < 0.01). Moreover, no significant correlation was found between 3-MCPDE levels and tumor stage or size in the RCC group. Conclusions: Accumulation of 3-MCPDEs in humans, with significantly higher levels was observed in kidney tumor specimens compared to non-patients. These findings suggest minimizing the intake of 3-MCPD and its esters in diets in order to reduce potential negative health impacts.

## 1. Introduction

3-Monochloro-propanediol esters (3-MCPDEs) constitute a class of contaminants prevalent in food processing, which primarily occur during the deodorization stage of oil refining [1,2,3]. Edible oils, particularly palm oil, are commonly consumed and employed as key ingredients in the production of processed foods such as cookies, instant noodles, and infant formula, contributing significantly to the heightened prevalence of 3-MCPDEs [4,5,6]. Research findings from previous investigations underscored that when subjected to high-temperature treatment (>200 °C) in the presence of chloride ions, oils and fats exhibit a notable capacity for the abundant production of 3-MCPDEs [2,3,4,7,8,9].

3-MCPDEs undergo hydrolysis, transforming into 3-monochloro-propanediol (3-MCPD) upon entering the biological system [2,3]. The kidneys and testes emerge as primary target organs for toxicity [2,3,10]. Experimental studies in Swiss mice and Sprague-Dawley (SD) rats, involving the administration of 3-MCPDEs, demonstrated correlations between exposure to these compounds and elevated levels of blood urea nitrogen (BUN) and creatinine (Cre) in serum. Renal pathologies induced by 3-MCPDE exposure encompass tubular necrosis, protein accumulation, and cell swelling degeneration [3,7,11,12]. Additionally, in the testes, 3-MCPDEs were shown to influence the testicular structure, giving rise to functional abnormalities such as the disruption of sperm maturation and impaired motility [13]. In a 2-year study, groups of 50 male and 50 female SD rats were exposed to 3-MCPD in their drinking water at different concentrations (0, 25, 100, or 400 ppm). Among male rats, there were dose-related increases in the occurrence of renal tubule adenomas or carcinomas and Leydig’s cell tumors, with significantly higher rates observed in the group exposed to 400 ppm of 3-MCPD. In female rats, there was a positive trend in the incidence of renal tubule adenomas, which became significant in the 400 ppm 3-MCPD group [14]. Although the carcinogenic evidence from animal experiments is still controversial, the International Agency for Research on Cancer (IARC) has classified 3-MCPD as a group 2B carcinogen, which are possibly carcinogenic to humans, with limited evidence of carcinogenicity in humans and insufficient evidence from animal studies [14,15,16].

Currently, there is no substantial evidence supporting the carcinogenicity of 3-MCPD in humans; only pooled estimates of total 3-MCPDE intake levels per day based on a questionnaire were compared between 77 renal cancer subjects and 80 matched controls to assess exposure and disease outcomes [17]. That study found no significant difference in 3-MCPDE intake between the cases and controls. Also, there was no association observed between high 3-MCPDE intake and the development of renal cancer. Herein, levels of 3-MCPDE accumulation in kidney tissues from both renal cell carcinoma (RCC) and non-RCC populations were directly analyzed for the purpose that this would contribute to a more precise comparison and a direct investigation into the association between 3-MCPDEs in organs and carcinogenicity.

## 2. Materials and Methods

### 2.1. Chemicals and Reagents

A Milli-Q water purification system (Millipore, Bedford, MA, USA) was used to produce ultrapure water. Toronto Research Chemicals (Toronto, ON, Canada) provided the analytical standards for rac-1,2-bis-palmitoyl-3-chloropropanediol (3-MCPD-PP) and dipalmito-yl-3-chloropropanediol-d5 (3-MCPD-PP-d5). J.T. Baker (Phillipsburg, NJ, USA) supplied all the analytical-grade chemicals for the experiment, and all the chemicals purchased from Sigma-Aldrich (St. Louis, MO, USA) were guaranteed to be of analytical-grade purity.

### 2.2. Patients and Data Collection

**Figure 1:**
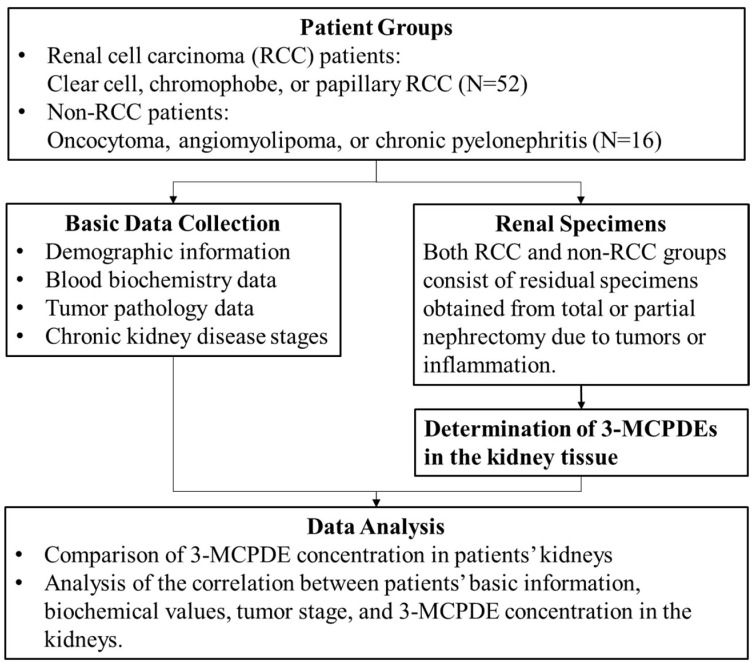
Experimental design and flowchart.

This study was carried out in accordance with a human subjects research protocol approved by the National Cheng Kung University Hospital Institutional Review Board (IRB) with no. A-ER-111-061 (Committee approval date: 12 May 2022). This study utilized residual specimens and medical records from kidney cancer patients enrolled in A-ER-106–451 from April 2018 to March 2022 at National Cheng Kung University Hospital, thereby exempting the need for informed consent from the participants. In total, 71 participants were initially recruited in A-ER-106-451 and provided informed consent. However, three participants had missing basic biochemical data and were subsequently excluded. As shown in Figure 1, the final analysis included 68 participants and focused on cumulative levels of 3-MCPDEs in their kidney tissues, along with their demographic and biochemical data. Participants were divided into two groups: an RCC patient group (*n* = 52) and a non-RCC patient group (*n* = 16). Patients in the RCC group underwent radical nephrectomy or partial nephrectomy with various approaches, including open surgery, laparoscopy, and robotic assistance. The non-RCC patient cohort comprised individuals who also underwent radical nephrectomy or partial nephrectomy and received a pathological diagnosis of renal angiomyolipoma, pyelonephritis, oncocytoma, or renal cysts. Residual renal tissue specimens were collected by obtaining a portion of kidney tissue during surgical procedures and were stored at −20 °C. Clinicopathologic data of patients were collected based on medical records, which included information such as the body mass index (BMI), estimated glomerular filtration rate (eGFR), aspartate aminotransferase (AST), alanine aminotransferase (ALT), tumor stage, tumor grade, and tumor size. The biochemical test items were sampled and analyzed by the Department of Pathology of National Cheng Kung University Hospital.

### 2.3. Method Validation

Eighteen-week-old rats (*n* = 5), considered adult rats, were used to simulate adult human kidneys to serve as a blank matrix in validation assays. The experimental subjects were 8-week-old male Sprague-Dawley (SD) rats purchased from Lesco BioTech Center (Taipei, Taiwan). They were fed 5001-Laboratory Rodent Diet for 10 weeks before being sacrificed for kidney analyses. Since the animal feed ingredients did not include edible oils or their products, rats were not exposed to 3-MCPDEs during their life.

The bioanalytical method was validated in terms of selectivity, sensitivity, linearity, precision, accuracy, and recovery according to the Food and Drug Administration (FDA)’s *Bioanalytical Method Validation Guidance for Industry* [18]. The selectivity of the method was routinely demonstrated by analyzing blank samples, which were rat kidney samples in this study to confirm that the assay was free of potential interfering substances. For sensitivity, the acceptable limit of quantification (LOQ) of 3-MCPDEs present in kidney samples was defined according to a signal-to-noise (S/N) ratio of ≥10 as 0.01 μg/g. Calibration curves with a concentration range of 0.01~10 μg/mL were prepared by dissolving 3-MCPD-PP in toluene, with an *R*^2^ value of >0.99. Five replicates utilizing rat kidney specimens spiked with a 3-MCPD-PP standard solution at two concentrations (0.05 and 0.25 µg/mL) were determined as quality control (QC) samples to perform assessments of intra-day and inter-day accuracy (recovery rate) and precision (coefficient of variance, CV). QC samples were analyzed in all analytical runs to meet the acceptance criteria of ±15% of nominal concentrations and ±15% CV set by guidance for bioanalytical method validation [18].

### 2.4. Determinations of 3-MCPDEs in Human Renal Samples

Renal specimens weighing 0.05 g we obtained from subjects were homogenized in 400 µL of ultrapure water and then centrifuged at 13,000× *g* for 10 min, followed by removal of the supernatant to remove potential 3-MCPD. The residual material was then mixed with 500 µL of n-hexane by agitation on a shaker for 10~15 s. Subsequently, samples were centrifuged at 300× *g* for 2 min, and the resulting supernatant was collected as the renal specimen extract. All sample preparation procedures and GC-MS analysis were carried out as described in the American Oil Chemists’ Society standard method Cd 29a-13 [6,19]. The samples were blinded for the lab workers. The renal specimen extract was transferred into a 10-mL glass tube with the addition of 50 μL of an internal standard solution (5 µg/mL 3-MCPD-PP-d_5_ corresponding to the free forms of 3-MCPD) and dissolved in 2 mL of THF. The mixture was homogenized and incubated at 50 °C for 15 min. GEs were converted to 3-monobromo-1,2-propanediol esters (3-MBPDEs) using a 30 μL volume of a NaBr acid aqueous solution (3.3 mg/mL, 5% H_2_SO_4_). After adding 3 mL of 0.6% NaHCO_3_ (*w*/*v*) to halt the reaction, 2 mL of n-heptane was used to extract the target chemical compounds. After transferring the upper layer to an empty glass tube and using a stream of N_2_ to evaporate it at 40 °C, the residue was dissolved in 1 mL of THF. The THF solution was mixed with 1.8 mL of a 1.8% sulfuric acid solution in methanol (*v*/*v*) to initiate the transesterification process, which was carried out for 16 h at 40 °C. A 0.5 mL volume of 9% NaHCO_3_ (*w*/*v*) was added to halt the reaction and the organic solvents evaporated at 40 °C under a N_2_ stream. After adding 2 mL of 20% Na_2_SO_4_ (*w*/*v*) to the sample, fatty acid methyl esters were extracted using 2 × 2 mL of heptane. Derivatization reactions were performed using 200 mL of a PBA solution (250 mg/mL, acetone: H_2_O 19:1, *v*/*v*) in an ultrasonic bath at room temperature for 5 min. N-heptane (2 × 1 mL) was used to extract PBA derivatives, which were then evaporated at 40 °C using a N_2_ stream. After dissolving the residue once again in 400 μL of n-heptane, the supernatant was transferred to a vial for GC-MS determination.

An Agilent 7820A GC with an Agilent 5977b inert single quadrupole MS (Agilent Technologies, Santa Clara, CA, USA) was used for the GC-MS analysis. A 1 μL sample extract was injected into a DB-5MS capillary column (30 m × 0.25 mm I.D. × 0.25 μm film thickness, Agilent Technologies) using pulsed splitless mode at 250 °C. The carrier gas employed was helium at a flow rate of 1.2 mL/min, with the transfer line temperature specifically set at 300 °C. The column temperature started at 80 °C for 1 min, then increased to 120 °C at 10 °C/min, followed by an increase to 156 °C at 3 °C/min, and finally reached 300 °C at 36 °C/min. The MS operated in electron ionization (EI) and selected ion monitoring (SIM) modes, detecting ions *m*/*z* 147 and 196 for 3-MCPD, and *m*/*z* 150 and 201 for the 3-MCPD-d5 internal standard. Results for 3-MCPDEs are presented in equimolar amounts of 3-MCPD.

### 2.5. Data Analysis

Microsoft Excel 2010 (Microsoft, Redmond, WA, USA) was used to record the concentrations of 3-MCPDEs in renal specimens as well as demographic data and biochemical markers. The values of non-detectable (ND) and <LOQ have been replaced by 0 and 0.005 μg/g, respectively. Mann–Whitney U-tests and Chi-squared tests were used to evaluate the differences between the groups. A Spearman correlation analysis was employed to investigate the correlation between basic information and biochemical values. Statistical significance was defined as *p* < 0.05, with *p* values between 0.05 and 0.1 considered marginally significant. The statistical analyses were performed with SPSS software (IBM version 19, Boston, MA, USA).

## 3. Results

### 3.1. Baseline Characteristics of RCC Patients and Non-RCC Patients

The basic demographic data of participants are summarized in Table 1. Among the 68 participants in this study, 45 (66.18%) were male and 23 (33.82%) were female. The median age was 63.5 years and the median body mass index (BMI) was 25.30 kg/m^2^. There were seven males (43.75%) and nine females (56.25%) in the non-RCC group, and 38 males (73.07%) and 16 females (26.93%) in the RCC group. A Chi-squared test was used to analyze gender distribution differences between the non-RCC and RCC groups. The results revealed a significantly higher proportion of males in the RCC group (*p* = 0.031). According to previous references, it is known that the male-to-female ratio of subjects with renal cell carcinoma is approximately 2:1 [20]. Age and BMI were analyzed using the Mann–Whitney U-test, and the results indicated no statistically significant differences between the groups (Table 1). There were also no statistically significant differences (*p* > 0.05) between the RCC and non-RCC groups in terms of the eGFR, chronic kidney disease (CKD) stage, or AST concentration in the blood. However, the blood ALT concentration in the RCC group was marginally higher than that in the non-RCC group, with a *p*-value of 0.063 (Table 1). Notably, the study’s constrained sample size of 68 participants, predominantly recruited from the southern region of Taiwan, necessitates caution in extrapolating the detection of 3-MCPDEs in kidney tumor tissues to represent the entire Taiwanese population.

### 3.2. Method Validation

As shown in Table 2, the mean intra-day recovery rates when spiked with 3-MCPD-PP equivalent concentrations of 0.05 and 0.25 µg/mL were 101.16% ± 8.75% and 105.00% ± 3.83%, respectively, and values of the intra-day precision (CV%) were 8.66% and 3.65%, respectively. Values of the inter-day accuracy for the same concentrations were 99.20% ± 3.92% and 100.72% ± 3.90%, respectively, with intra-day precisions of 3.95% and 3.88%, respectively. Recovery rates and CV% at these two concentrations met the specifications of 80~120% and <20% in guidelines for bioanalytical method validation [18]. These intra- and inter-day precision results demonstrated that the approach was representative and robust for sample analysis. Previously, the AOCS Cd 29a-13 method was implemented to determine 3-MCPDEs in edible oils, foodstuffs, and milk powder [4,5,6]. This study validated a sample preparation method for the analysis of 3-MCPDEs in renal specimens for the first time.

### 3.3. Analysis of Cumulative 3-MCPDE Levels in Kidney Tissues

In the non-RCC group, only one individual (6.25%) had detectable 3-MCPDEs in their kidney tissues. The average 3-MCPDE accumulation in the non-RCC group was 0.01 ± 0.02 µg/g. In the RCC group, 29 participants (55.76%) had detectable 3-MCPDEs in their kidney tissues, with an average 3-MCPDE accumulation of 0.22 ± 0.45 µg/g. The range of accumulation levels spanned from ND to 2.22 µg/g. 3-MCPDE accumulation in the RCC group was statistically significantly higher than that in the non-RCC group (*p* < 0.01) (Table 3). 3-MCPDE accumulation in females was 0.11 ± 0.20 µg/g, while in males it was 0.27 ± 0.50 µg/g. There was no statistically significant difference between males and females (*p* > 0.05).

### 3.4. Analysis of Correlations between Cumulative 3-MCPDE Levels in Kidney Tissues and Basic Demographic Data and Biochemical Indicators

We utilized Spearman’s correlation coefficients to analyze correlations between cumulative 3-MCPDE levels in kidney tissues and basic demographic data, as shown in Table 4. Cumulative 3-MCPDE levels in kidney tissues exhibited a marginally significant positive correlation with blood ALT concentration (*r* = 0.236, *p* = 0.06), but did not show statistically significant correlations with gender, age, BMI, eGFR, CKD stage, blood AST concentrations, tumor size, or tumor grade (*p* > 0.05). Animal experiments demonstrated hepatotoxicity associated with 3-MCPDEs, characterized by pathological changes in liver tissues, including swelling, inflammatory cell infiltration, and fat accumulation [2,21,22]. Limited epidemiological data and research on the hepatotoxicity of 3-MCPDEs in humans warrant further investigation to elucidate the underlying mechanisms.

## 4. Discussion

Our study presents pioneering research revealing the accumulation of 3-MCPDEs in human kidney tissues, exhibiting significantly elevated levels in renal tissues of RCC patients compared to their non-RCC counterparts (*p* < 0.05). These findings suggest the bioavailability of dietary 3-MCPDEs, signifying their absorption by the human body and subsequent distribution throughout various organs and tissues. Upon ingestion, although 3-MCPDEs traverse the gastrointestinal tract and undergo hydrolysis into 3-MCPD through the action of lipase, re-esterification and binding with endogenous compounds could occur in the liver and kidneys [23,24]. In rats administered a dose of 3-MCPD-PP at 53.2 mg/kg body weight (BW), 3-MCPDEs were detected in the blood, intestines, liver, kidneys, and fat tissues at varying time intervals (1.5, 3, 6, 12, and 24 h), with the highest recovery rate observed in the kidneys at 3~6 h post-exposure. The recovery rate gradually declined over time, reaching 0.002% at 24 h post-exposure. Excretion patterns indicated that more hydrophobic metabolites were excreted in feces, while more hydrophilic ones were excreted in urine. There is currently limited research on the distribution of 3-MCPDEs in human organ tissues, with only a few studies detecting 3-MCPDE in human breast milk [25,26,27], aligning with results of our study, with both indicating the presence of 3-MCPD in ester form in the human body. Nevertheless, it was reported that after consumption of hazelnut oil containing 3-MCPDEs, the excretion of 3-MCPD in urine significantly increased, with approximately 3.7% excretion [28]. The excretion of metabolized 3-MCPDEs may decrease in the presence of renal tumors. With prolonged dietary exposure, a certain extent of 3-MCPDEs that cannot be eliminated may accumulate in the kidneys, which could serve as a potential biomarker.

In vitro research has shown that 3-MCPD can cause significant damage to various cell types, including rat Leydig cells, rat kidney proximal tubular epithelial cells, and human embryonic kidney cells [3]. This damage is characterized by the activation of mitochondrial apoptotic pathways and death receptor pathways, which are crucial for regulating cell death and proliferation [3]. Additionally, gene expression analyses indicate that 3-MCPD disrupts the regulation of apoptotic genes, potentially affecting normal cell growth and survival mechanisms [3]. Cytotoxicity assays further corroborate these findings, demonstrating that 3-MCPD is particularly harmful to rat kidney cells, resulting in dose-dependent weight loss and kidney damage [3]. Previous animal experiments have indicated a potential association between exposure to 3-MCPD and renal carcinogenesis [14,16]. One in vivo study reported that administering 3-MCPD in drinking water to rats increased the incidence of renal tubule carcinoma, renal tubule adenoma or carcinoma (combined), and Leydig cell adenoma in males, as well as renal tubule adenoma or carcinoma (combined) in females [14]. In another study, administration by gavage to rats did not increase tumor incidence [16]. The discrepancies between the in vivo results underscore that the carcinogenic risk of 3-MCPDEs may be context-dependent, influenced by factors such as dosage and exposure duration. Long-term, high-dose exposure could potentially increase cancer risk, particularly in tissues vulnerable to oxidative stress and inflammation.

Given the multifaceted nature of RCC carcinogenesis, our study exclusively explored the link between 3-MCPDEs and RCC without examining other contributing factors. It serves as preliminary research on the accumulation of 3-MCPDEs in organs and its potential association with carcinogenicity. Furthermore, no significant correlations were observed between the accumulation of 3-MCPDEs in kidney tissues and tumor stage or size, suggesting that 3-MCPDEs, as a potential risk factor for RCC, may be related more to the occurrence of RCC rather than to its development process.

The limitations of this study include a smaller sample size for the control group due to the difficulty in obtaining specimens from such patients. Additionally, this is a cross-sectional study. It is hypothesized that kidney cancer patients may have ingested higher amounts of 3-MCPDEs, which could contribute to disease onset, or their reduced excretion efficiency leads to accumulation. Future research should investigate the correlation between 3-MCPDE intake and contamination levels, focusing on the chronic effects of long-term dietary exposure on renal cancer incidence and renal tumor progression in high-risk populations.

## 5. Conclusions

This study represents the first assessment of 3-monochloropropanediol ester (3-MCPDE) accumulation in human kidney tissues, focusing on a cohort of 68 individuals, including those with and without renal cell carcinoma (RCC). Our investigation employed a partially validated analytical method for quantifying 3-MCPDEs in kidney tissues. The results demonstrated significantly elevated levels of 3-MCPDEs in kidney tissues from RCC patients compared to non-RCC participants. However, no significant correlation was observed between 3-MCPDE levels and tumor stage or size within the RCC group. The pronounced accumulation of 3-MCPDEs in kidney tissues, particularly in tumor specimens, suggests a potential link between 3-MCPDEs and RCC.

## Figures and Tables

**Table 1:** Baseline characteristics of renal cell carcinoma (RCC) patients and non-RCC patients.

	All (*n* = 68)	Non-RCC (*n* = 16)	RCC (*n* = 52)
	*n* (%)/Median (Range)		
Sex, male	45 (66.18%)	7 (43.75%)	38 (73.07%) *
Age (years)	63.5 (32~88)	60 (32~81)	65.5 (39~88)
BMI (kg/m^2^)	25.3 (17.47~40)	24.44 (19.50~40)	26.36 (17.47~35.95)
eGFR (mL/min/1.73 m^2^)	87.1 (6.13~183.61)	86.4 (6.13~166.31)	88.04 (6.6~183.61)
CKD stage	2 (1~5)	2 (1~5)	2 (1~5)
AST (U/L)	25 (12~122)	23 (15~118)	25.5 (12~122)
ALT (U/L)	22 (5~199)	15 (5~85)	26 (10~199)
Tumor size (cm^3^)	-	-	21.9 (3~931)
Tumor grade	-	-	2 (1~4)

* *p* < 0.05; RCC, renal cell carcinoma; BMI, body mass index; eGFR, estimated glomerular filtration rate; CKD, chronic kidney disease; AST, aspartate aminotransferase; ALT, alanine aminotransferase.

**Table 2:** Evaluation of intra-day and inter-day precisions of the analytical method.

3-MCPD-PP Equivalent Concentration (µg/mL)		Recovery (%)	RSD (%)
0.05	Intra-day	101.16 ± 8.75	8.66
	Inter-day	99.20 ± 3.92	3.95
0.25	Intra-day	105.00 ± 3.83	3.65
	Inter-day	100.72 ± 3.90	3.88

RSD, relative standard deviation.

**Table 3:** Distribution of cumulative 3-monochloro-propanediol ester (3-MCPDE) levels in kidney tissues of study patients.

	*n*	3-MCPDE Accumulation (µg/g)		Detection Rate
		Mean ± SD	Range	
RCC	52	0.22 ± 0.45	ND~2.22 **	55.76%
Non-RCC	16	0.01 ± 0.02	ND~0.10	6.25%

** *p* < 0.01. RCC, renal cell carcinoma; 3-MCPDE, 3-monochloro-propanediol ester; SD, standard deviation.

**Table 4:** Spearman’s correlation coefficient analysis of the cumulative 3-monochloro-propanediol ester (3-MCPDE) levels in kidney tissues of renal cell carcinoma (RCC) patients in relation to basic demographic data and basic biochemical indicators.

	3-MCPDEs Accumulation	Sex	Age	BMI	eGFR	CKD Stage	AST	ALT	Tumor Size	Tumor Grade
3-MCPDEs accumulation	1									
Sex	0.174	1								
Age	0.086	−0.208	1							
BMI	0.178	−0.087	−0.11	1						
eGFR	−0.208	−0.257 ^#^	−0.216	0.151	1					
CKD stage	0.199	0.249 ^#^	0.267 ^#^	−0.19	−0.931 **	1				
AST	0.192	0.09	−0.025	−0.062	0.312 *	−0.328 *	1			
ALT	0.236 ^#^	0.249 ^#^	−0.12	0.151	0.283 *	−0.257 ^#^	0.792 **	1		
Tumor size	−0.069	−0.149	0.228 ^#^	−0.158	0.143	−0.119	−0.388 **	−0.364 **	1	
Tumor grade	0.127	0.263 ^#^	−0.061	−0.261 ^#^	0.085	−0.08	0.13	0.058	0.232 ^#^	1

^#^: *p* < 0.1, *: *p* < 0.05, **: *p* < 0.01. 3-MCPDEs, 3-monochloro-propanediol esters; BMI, body mass index; eGFR, estimated glomerular filtration rate; CKD, chronic kidney disease; AST, aspartate aminotransferase; ALT, alanine aminotransferase.

## Data Availability

The data that support the findings of this study are available from the corresponding author upon reasonable request.
